# 5-(Adamantan-1-yl)-3-anilinomethyl-2,3-di­hydro-1,3,4-oxa­diazole-2-thione

**DOI:** 10.1107/S1600536813009835

**Published:** 2013-04-13

**Authors:** Abdul-Malek S. Al-Tamimi, Omar A. Al-Deeb, Ali A. El-Emam, Seik Weng Ng, Edward R. T. Tiekink

**Affiliations:** a Department of Pharmaceutical Chemistry, College of Pharmacy, Salman bin Abdulaziz University, Alkharj 11942, Saudi Arabia; b Department of Pharmaceutical Chemistry, College of Pharmacy, King Saud University, Riyadh 11451, Saudi Arabia; c Department of Chemistry, University of Malaya, 50603 Kuala Lumpur, Malaysia; d Chemistry Department, Faculty of Science, King Abdulaziz University, PO Box 80203 Jeddah, Saudi Arabia

## Abstract

In the title compound, C_19_H_23_N_3_OS, the oxa­diazole and benzene rings are inclined at a dihedral angle of 50.30 (11)°, with the major twist between them occurring at the ring–methyl­ene N—C bond [N—N—C—N torsion angle = −101.2 (2)°]. In the crystal, helical supra­molecular chains along [010] are sustained by N—H⋯S hydrogen bonds. These are linked into layers lying parallel to (-101) by methyl­ene–phenyl C—H⋯π inter­actions.

## Related literature

For the anti-viral and anti-inflammatory activity of adamantane derivatives, see: El-Emam *et al.* (2004 ▶); El-Emam & Ibrahim (1991 ▶). For the structure of the 4-fluoro derivative, see: Al-Tamimi *et al.* (2013 ▶).
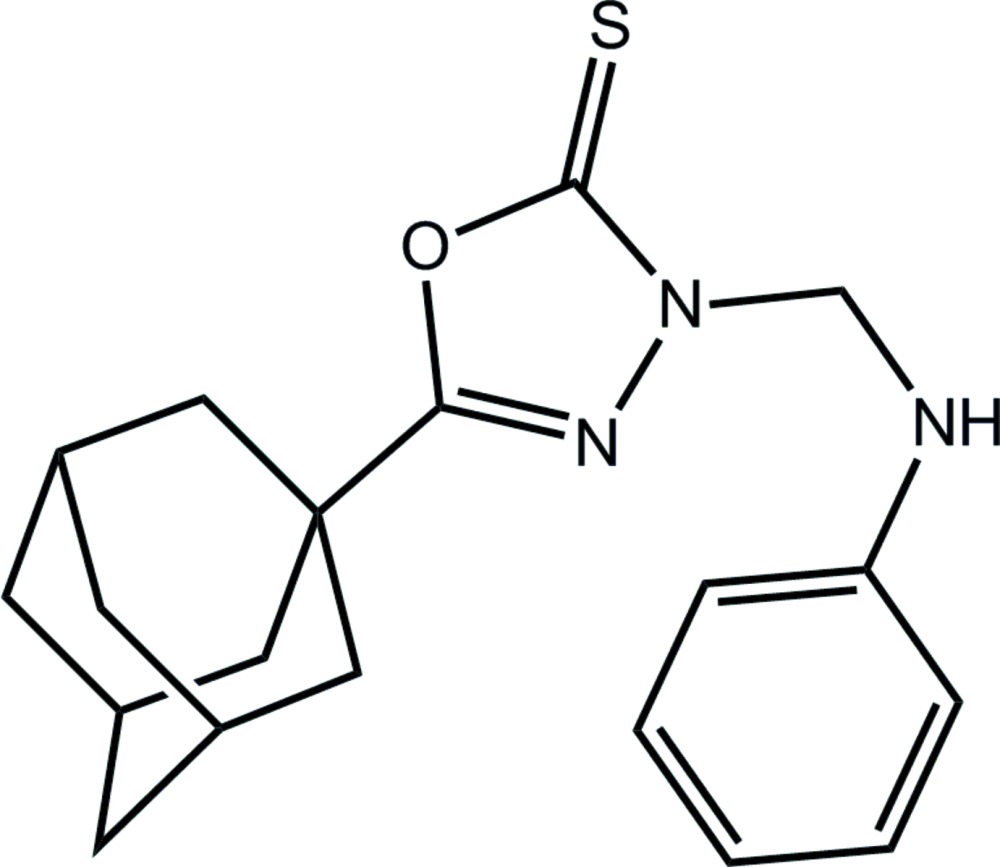


## Experimental

### 

#### Crystal data

C_19_H_23_N_3_OS
*M* *_r_* = 341.46
Monoclinic, 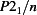

*a* = 14.1326 (13) Å
*b* = 7.1179 (5) Å
*c* = 18.3685 (16) Å
β = 105.546 (10)°
*V* = 1780.2 (3) Å^3^
*Z* = 4
Mo *K*α radiation
μ = 0.19 mm^−1^
*T* = 295 K
0.40 × 0.30 × 0.20 mm

#### Data collection

Agilent SuperNova Dual diffractometer with an Atlas detector
Absorption correction: multi-scan (*CrysAlis PRO*; Agilent, 2011 ▶) *T*_min_ = 0.863, *T*_max_ = 1.000
12073 measured reflections
4115 independent reflections
2827 reflections with *I* > 2σ(*I*)
*R*_int_ = 0.033

#### Refinement

*R*[*F* ^2^ > 2σ(*F* ^2^)] = 0.047
*wR*(*F* ^2^) = 0.131
*S* = 1.03
4115 reflections
221 parameters
1 restraint
H atoms treated by a mixture of independent and constrained refinement
Δρ_max_ = 0.27 e Å^−3^
Δρ_min_ = −0.21 e Å^−3^

### 

Data collection: *CrysAlis PRO* (Agilent, 2011 ▶); cell refinement: *CrysAlis PRO*; data reduction: *CrysAlis PRO*; program(s) used to solve structure: *SHELXS97* (Sheldrick, 2008 ▶); program(s) used to refine structure: *SHELXL97* (Sheldrick, 2008 ▶); molecular graphics: *ORTEP-3 for Windows* (Farrugia, 2012 ▶) and *DIAMOND* (Brandenburg, 2006 ▶); software used to prepare material for publication: *publCIF* (Westrip, 2010 ▶).

## Supplementary Material

Crystal structure: contains datablock(s) global, I. DOI: 10.1107/S1600536813009835/hb7068sup1.cif

Structure factors: contains datablock(s) I. DOI: 10.1107/S1600536813009835/hb7068Isup2.hkl

Supplementary material file. DOI: 10.1107/S1600536813009835/hb7068Isup3.cml

Additional supplementary materials:  crystallographic information; 3D view; checkCIF report

## Figures and Tables

**Table 1:** Hydrogen-bond geometry (Å, °) *Cg*1 is the centroid of the C14–C19 ring.

*D*—H⋯*A*	*D*—H	H⋯*A*	*D*⋯*A*	*D*—H⋯*A*
N3—H3⋯S1^i^	0.87 (1)	2.62 (1)	3.4763 (19)	170 (2)
C13—H13*A*⋯*Cg*1^ii^	0.97	2.83	3.549 (2)	132

Symmetry codes: (i) 
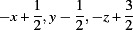
; (ii) 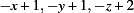.

**Fig. 1.**
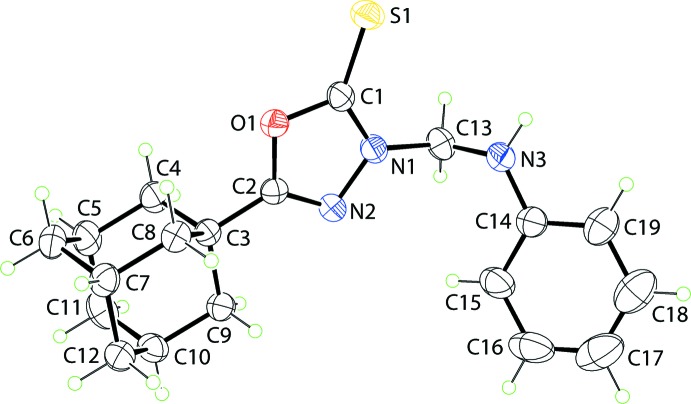
The molecular structure of (I) showing displacement ellipsoids at the 35% probability level.

**Fig. 2.**
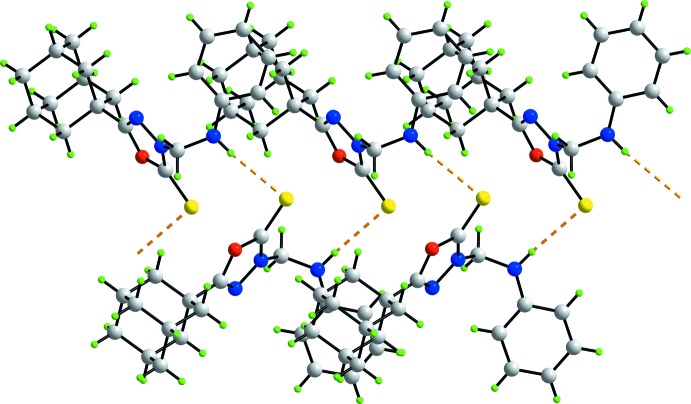
A view of the helical supramolecular chain along the b axis in (I), which is sustained by N—H···S hydrogen bonds shown as orange dashed lines.

**Fig. 3.**
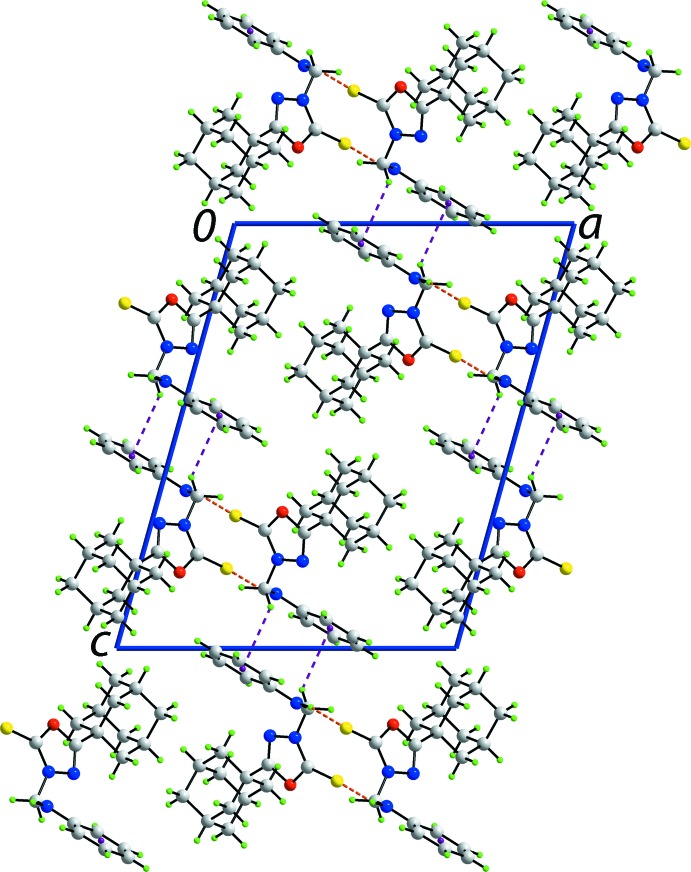
View of the unit-cell contents in projection down the b axis of (I). The N—H···S and C—H···π interactions are shown as orange and purple dashed lines, respectively.

## supplementary crystallographic information

### Comment

In continuation of our long-term interest in the chemical and pharmacological properties of adamantane derivatives (El-Emam & Ibrahim, 1991; El-Emam *et al.*, 2004), including structural studies (Al-Tamimi *et al.*, 2013), we describe herein the X-ray crystal structure determination of the title compound, (I).

In (I), Fig. 1, the oxadiazole ring is strictly planar (r.m.s. deviation = 0.003 Å) and the thione-S1 atom lies in this plane. The benzene ring is highly twisted out of this plane, forming a dihedral angle of 50.30 (11)°; the N2—N1—C13—N3 torsion angle = -101.2 (2)°. Despite the kink in the molecule, the thione-S1 and amine-N3—H atoms are *syn*, as seen in the S1—C1···N3—H3 torsion angle of 3 (1)°. This allows for the formation of N—H···S hydrogen bonds that lead to helical supramolecular chains along the *b* axis, Fig. 2 and Table 1. Chains are connected into layers parallel to (101) by methylene-C—H···π(phenyl) interactions, Fig. 3 and Table 1.

### Experimental

A mixture of 5-(adamantane-1-yl)-1,3,4-oxadiazole-2-thiol (2.36 g, 0.01 mol), aniline (0.93 g, 0.01 mol) and 37% formaldehyde solution (1.5 ml), in ethanol (15 ml), was stirred at room temperature for 2 h and allowed to stand overnight. The precipitated crude product was filtered, washed with water, dried, and crystallized from ethanol to yield 3.04 g (89%) of the title compound (I) as fine colourless crystals. *M*.pt: 439–471 K. Colourless prisms were obtained by slow evaporation of its CHCl_3_-ethanol (1:1; 10 ml) solution held at room temperature. ^1^H NMR (CDCl_3_, 500.13 MHz): δ 1.75 (*q*, 6H, adamantane-H), 2.01 (*s*, 6H, adamantane-H), 2.12 (*s*, 3H, adamantane-H), 2.98 (*t*, 4H, piperazine-H), 3.20 (*t*, 4H, piperazine-H), 5.06 (*s*, 2H, CH_2_), 6.88–6.95 (*m*, 3H, Ar—H), 7.27 (*t*, 2H, Ar—H, J = 7.5 Hz). ^13^C NMR (CDCl_3_, 125.76 MHz): δ 27.47, 34.40, 36.10, 39.12 (adamantane-C), 49.36, 50.26 (piperazine-C), 69.86 (CH_2_), 116.43, 120.15, 129.17, 151.22 (Ar—C), 167.91 (C═N), 178.62 (C═S).

### Refinement

The H-atoms were placed in calculated positions [C—H = 0.93 to 0.98 Å, *U*_iso_(H) = 1.2*U*_eq_(C)] and were included in the refinement in the riding model approximation. The N-bound H-atom was refined with the distance restraint N—H = 0.88±0.01 Å.

### Crystal data

**Table:**

C_19_H_23_N_3_OS	*F*(000) = 728
*M**_r_* = 341.46	*D*_x_ = 1.274 Mg m^−^^3^
Monoclinic, *P*2_1_/*n*	Mo *K*α radiation, λ = 0.71073 Å
Hall symbol: -P 2yn	Cell parameters from 2861 reflections
*a* = 14.1326 (13) Å	θ = 2.9–27.5°
*b* = 7.1179 (5) Å	µ = 0.19 mm^−^^1^
*c* = 18.3685 (16) Å	*T* = 295 K
β = 105.546 (10)°	Prism, colourless
*V* = 1780.2 (3) Å^3^	0.40 × 0.30 × 0.20 mm
*Z* = 4	

### Data collection

**Table:**

Agilent SuperNova Dual diffractometer with an Atlas detector	4115 independent reflections
Radiation source: SuperNova (Mo) X-ray Source	2827 reflections with *I* > 2σ(*I*)
Mirror monochromator	*R*_int_ = 0.033
Detector resolution: 10.4041 pixels mm^-1^	θ_max_ = 27.6°, θ_min_ = 3.0°
ω scan	*h* = −16→18
Absorption correction: multi-scan (*CrysAlis PRO*; Agilent, 2011)	*k* = −7→9
*T*_min_ = 0.863, *T*_max_ = 1.000	*l* = −23→22
12073 measured reflections	

### Refinement

**Table:**

Refinement on *F*^2^	Primary atom site location: structure-invariant direct methods
Least-squares matrix: full	Secondary atom site location: difference Fourier map
*R*[*F*^2^ > 2σ(*F*^2^)] = 0.047	Hydrogen site location: inferred from neighbouring sites
*wR*(*F*^2^) = 0.131	H atoms treated by a mixture of independent and constrained refinement
*S* = 1.03	*w* = 1/[σ^2^(*F*_o_^2^) + (0.0529*P*)^2^ + 0.3647*P*] where *P* = (*F*_o_^2^ + 2*F*_c_^2^)/3
4115 reflections	(Δ/σ)_max_ < 0.001
221 parameters	Δρ_max_ = 0.27 e Å^−^^3^
1 restraint	Δρ_min_ = −0.21 e Å^−^^3^

### Special details

**Table:**

Geometry. All e.s.d.'s (except the e.s.d. in the dihedral angle between two l.s. planes) are estimated using the full covariance matrix. The cell e.s.d.'s are taken into account individually in the estimation of e.s.d.'s in distances, angles and torsion angles; correlations between e.s.d.'s in cell parameters are only used when they are defined by crystal symmetry. An approximate (isotropic) treatment of cell e.s.d.'s is used for estimating e.s.d.'s involving l.s. planes.
Refinement. Refinement of *F*^2^ against ALL reflections. The weighted *R*-factor *wR* and goodness of fit *S* are based on *F*^2^, conventional *R*-factors *R* are based on *F*, with *F* set to zero for negative *F*^2^. The threshold expression of *F*^2^ > σ(*F*^2^) is used only for calculating *R*-factors(gt) *etc*. and is not relevant to the choice of reflections for refinement. *R*-factors based on *F*^2^ are statistically about twice as large as those based on *F*, and *R*- factors based on ALL data will be even larger.

### Fractional atomic coordinates and isotropic or equivalent isotropic displacement parameters (Å^2^)

**Table:**

	*x*	*y*	*z*	*U*_iso_*/*U*_eq_	
S1	0.24181 (4)	0.53985 (8)	0.68790 (3)	0.05916 (19)	
O1	0.37654 (9)	0.79478 (17)	0.67903 (7)	0.0445 (3)	
N1	0.40128 (11)	0.6798 (2)	0.79036 (8)	0.0443 (4)	
N2	0.47627 (11)	0.8094 (2)	0.79447 (8)	0.0439 (4)	
N3	0.42471 (14)	0.4044 (3)	0.87149 (10)	0.0572 (5)	
H3	0.3797 (12)	0.317 (2)	0.8616 (12)	0.064 (7)*	
C1	0.33997 (14)	0.6695 (2)	0.72114 (10)	0.0434 (4)	
C2	0.45822 (13)	0.8739 (2)	0.72695 (9)	0.0387 (4)	
C3	0.51147 (13)	1.0228 (2)	0.69660 (9)	0.0380 (4)	
C4	0.44544 (15)	1.1970 (3)	0.67713 (12)	0.0521 (5)	
H4A	0.3850	1.1644	0.6397	0.063*	
H4B	0.4288	1.2415	0.7220	0.063*	
C5	0.49874 (17)	1.3518 (3)	0.64617 (13)	0.0600 (6)	
H5	0.4563	1.4627	0.6340	0.072*	
C6	0.52324 (16)	1.2811 (3)	0.57462 (12)	0.0601 (6)	
H6A	0.4632	1.2484	0.5368	0.072*	
H6B	0.5562	1.3793	0.5541	0.072*	
C7	0.58959 (15)	1.1093 (3)	0.59392 (10)	0.0499 (5)	
H7	0.6054	1.0647	0.5481	0.060*	
C8	0.53678 (15)	0.9534 (3)	0.62449 (10)	0.0445 (4)	
H8A	0.5786	0.8433	0.6360	0.053*	
H8B	0.4771	0.9188	0.5867	0.053*	
C9	0.60678 (14)	1.0759 (3)	0.75559 (9)	0.0445 (4)	
H9A	0.5919	1.1190	0.8014	0.053*	
H9B	0.6490	0.9665	0.7680	0.053*	
C10	0.65936 (15)	1.2308 (3)	0.72437 (11)	0.0516 (5)	
H10	0.7201	1.2644	0.7624	0.062*	
C11	0.59323 (18)	1.4034 (3)	0.70569 (13)	0.0638 (6)	
H11A	0.6270	1.5030	0.6867	0.077*	
H11B	0.5777	1.4484	0.7510	0.077*	
C12	0.68424 (15)	1.1618 (3)	0.65259 (11)	0.0525 (5)	
H12A	0.7180	1.2601	0.6329	0.063*	
H12B	0.7272	1.0534	0.6642	0.063*	
C13	0.38778 (16)	0.5893 (3)	0.85927 (11)	0.0539 (5)	
H13A	0.4199	0.6655	0.9026	0.065*	
H13B	0.3182	0.5869	0.8562	0.065*	
C14	0.51825 (16)	0.3569 (3)	0.91240 (10)	0.0501 (5)	
C15	0.59359 (18)	0.4873 (4)	0.93158 (12)	0.0721 (7)	
H15	0.5821	0.6127	0.9178	0.087*	
C16	0.6873 (2)	0.4277 (6)	0.97194 (15)	0.0973 (11)	
H16	0.7381	0.5145	0.9856	0.117*	
C17	0.7051 (2)	0.2421 (6)	0.99156 (16)	0.1006 (11)	
H17	0.7679	0.2037	1.0178	0.121*	
C18	0.6307 (2)	0.1144 (5)	0.97258 (14)	0.0864 (9)	
H18	0.6427	−0.0112	0.9858	0.104*	
C19	0.53828 (19)	0.1709 (3)	0.93402 (11)	0.0643 (6)	
H19	0.4878	0.0830	0.9220	0.077*	

### Atomic displacement parameters (Å^2^)

**Table:**

	*U*^11^	*U*^22^	*U*^33^	*U*^12^	*U*^13^	*U*^23^
S1	0.0456 (3)	0.0447 (3)	0.0819 (4)	−0.0051 (2)	0.0079 (3)	0.0002 (2)
O1	0.0420 (7)	0.0413 (7)	0.0474 (7)	−0.0013 (6)	0.0072 (6)	0.0018 (5)
N1	0.0449 (9)	0.0411 (8)	0.0483 (8)	−0.0022 (8)	0.0147 (7)	0.0023 (6)
N2	0.0435 (9)	0.0417 (8)	0.0467 (8)	−0.0038 (8)	0.0126 (7)	0.0011 (7)
N3	0.0549 (11)	0.0478 (10)	0.0605 (10)	−0.0125 (9)	0.0007 (9)	0.0073 (8)
C1	0.0428 (10)	0.0340 (9)	0.0558 (11)	0.0045 (8)	0.0171 (9)	0.0021 (8)
C2	0.0375 (10)	0.0371 (9)	0.0412 (9)	0.0024 (8)	0.0101 (8)	−0.0024 (7)
C3	0.0389 (10)	0.0354 (9)	0.0403 (9)	0.0024 (8)	0.0115 (8)	−0.0006 (7)
C4	0.0488 (12)	0.0452 (11)	0.0661 (12)	0.0104 (10)	0.0217 (10)	0.0070 (9)
C5	0.0618 (14)	0.0390 (10)	0.0846 (15)	0.0146 (10)	0.0291 (12)	0.0150 (10)
C6	0.0578 (13)	0.0620 (13)	0.0601 (12)	0.0056 (11)	0.0154 (11)	0.0230 (10)
C7	0.0545 (12)	0.0577 (12)	0.0401 (9)	0.0046 (10)	0.0173 (9)	0.0026 (8)
C8	0.0491 (11)	0.0428 (10)	0.0412 (9)	0.0030 (9)	0.0117 (8)	−0.0023 (7)
C9	0.0454 (11)	0.0485 (11)	0.0400 (9)	−0.0011 (9)	0.0117 (8)	−0.0012 (8)
C10	0.0492 (11)	0.0535 (12)	0.0508 (11)	−0.0106 (10)	0.0115 (9)	−0.0037 (9)
C11	0.0811 (17)	0.0411 (11)	0.0773 (14)	−0.0083 (12)	0.0352 (13)	−0.0054 (10)
C12	0.0461 (11)	0.0593 (12)	0.0550 (11)	0.0023 (10)	0.0188 (9)	0.0079 (9)
C13	0.0588 (13)	0.0555 (12)	0.0532 (11)	−0.0052 (11)	0.0250 (10)	0.0048 (9)
C14	0.0522 (12)	0.0644 (13)	0.0340 (9)	−0.0061 (11)	0.0121 (9)	0.0013 (8)
C15	0.0603 (15)	0.0996 (19)	0.0548 (12)	−0.0252 (14)	0.0124 (11)	0.0134 (12)
C16	0.0567 (17)	0.168 (3)	0.0676 (16)	−0.032 (2)	0.0168 (14)	0.0173 (19)
C17	0.0655 (19)	0.173 (4)	0.0645 (16)	0.026 (2)	0.0193 (15)	0.025 (2)
C18	0.093 (2)	0.106 (2)	0.0594 (14)	0.032 (2)	0.0173 (15)	0.0027 (14)
C19	0.0756 (16)	0.0685 (15)	0.0453 (11)	0.0103 (13)	0.0101 (11)	−0.0020 (10)

### Geometric parameters (Å, º)

**Table:**

S1—C1	1.642 (2)	C8—H8A	0.9700
O1—C1	1.369 (2)	C8—H8B	0.9700
O1—C2	1.371 (2)	C9—C10	1.525 (3)
N1—C1	1.335 (2)	C9—H9A	0.9700
N1—N2	1.392 (2)	C9—H9B	0.9700
N1—C13	1.478 (2)	C10—C11	1.526 (3)
N2—C2	1.283 (2)	C10—C12	1.533 (3)
N3—C14	1.376 (3)	C10—H10	0.9800
N3—C13	1.411 (3)	C11—H11A	0.9700
N3—H3	0.872 (10)	C11—H11B	0.9700
C2—C3	1.492 (2)	C12—H12A	0.9700
C3—C9	1.533 (3)	C12—H12B	0.9700
C3—C4	1.535 (2)	C13—H13A	0.9700
C3—C8	1.543 (2)	C13—H13B	0.9700
C4—C5	1.528 (3)	C14—C15	1.385 (3)
C4—H4A	0.9700	C14—C19	1.389 (3)
C4—H4B	0.9700	C15—C16	1.399 (4)
C5—C11	1.527 (3)	C15—H15	0.9300
C5—C6	1.531 (3)	C16—C17	1.375 (5)
C5—H5	0.9800	C16—H16	0.9300
C6—C7	1.524 (3)	C17—C18	1.363 (4)
C6—H6A	0.9700	C17—H17	0.9300
C6—H6B	0.9700	C18—C19	1.368 (3)
C7—C12	1.522 (3)	C18—H18	0.9300
C7—C8	1.526 (3)	C19—H19	0.9300
C7—H7	0.9800		
			
C1—O1—C2	106.57 (13)	H8A—C8—H8B	108.2
C1—N1—N2	112.24 (14)	C10—C9—C3	109.68 (15)
C1—N1—C13	126.30 (16)	C10—C9—H9A	109.7
N2—N1—C13	120.92 (15)	C3—C9—H9A	109.7
C2—N2—N1	103.52 (15)	C10—C9—H9B	109.7
C14—N3—C13	125.16 (19)	C3—C9—H9B	109.7
C14—N3—H3	118.6 (15)	H9A—C9—H9B	108.2
C13—N3—H3	114.4 (15)	C9—C10—C11	109.63 (16)
N1—C1—O1	104.80 (15)	C9—C10—C12	109.81 (16)
N1—C1—S1	130.78 (14)	C11—C10—C12	109.40 (16)
O1—C1—S1	124.43 (14)	C9—C10—H10	109.3
N2—C2—O1	112.87 (15)	C11—C10—H10	109.3
N2—C2—C3	128.79 (16)	C12—C10—H10	109.3
O1—C2—C3	118.31 (14)	C10—C11—C5	109.43 (17)
C2—C3—C9	110.28 (14)	C10—C11—H11A	109.8
C2—C3—C4	108.94 (14)	C5—C11—H11A	109.8
C9—C3—C4	109.27 (15)	C10—C11—H11B	109.8
C2—C3—C8	110.47 (14)	C5—C11—H11B	109.8
C9—C3—C8	108.90 (14)	H11A—C11—H11B	108.2
C4—C3—C8	108.96 (14)	C7—C12—C10	109.08 (15)
C5—C4—C3	109.84 (15)	C7—C12—H12A	109.9
C5—C4—H4A	109.7	C10—C12—H12A	109.9
C3—C4—H4A	109.7	C7—C12—H12B	109.9
C5—C4—H4B	109.7	C10—C12—H12B	109.9
C3—C4—H4B	109.7	H12A—C12—H12B	108.3
H4A—C4—H4B	108.2	N3—C13—N1	114.57 (16)
C4—C5—C11	109.33 (17)	N3—C13—H13A	108.6
C4—C5—C6	109.28 (17)	N1—C13—H13A	108.6
C11—C5—C6	109.76 (18)	N3—C13—H13B	108.6
C4—C5—H5	109.5	N1—C13—H13B	108.6
C11—C5—H5	109.5	H13A—C13—H13B	107.6
C6—C5—H5	109.5	N3—C14—C15	122.3 (2)
C7—C6—C5	109.30 (16)	N3—C14—C19	118.8 (2)
C7—C6—H6A	109.8	C15—C14—C19	118.8 (2)
C5—C6—H6A	109.8	C14—C15—C16	119.0 (3)
C7—C6—H6B	109.8	C14—C15—H15	120.5
C5—C6—H6B	109.8	C16—C15—H15	120.5
H6A—C6—H6B	108.3	C17—C16—C15	120.7 (3)
C12—C7—C6	109.66 (17)	C17—C16—H16	119.6
C12—C7—C8	109.99 (15)	C15—C16—H16	119.6
C6—C7—C8	109.76 (15)	C18—C17—C16	120.0 (3)
C12—C7—H7	109.1	C18—C17—H17	120.0
C6—C7—H7	109.1	C16—C17—H17	120.0
C8—C7—H7	109.1	C17—C18—C19	120.0 (3)
C7—C8—C3	109.39 (14)	C17—C18—H18	120.0
C7—C8—H8A	109.8	C19—C18—H18	120.0
C3—C8—H8A	109.8	C18—C19—C14	121.4 (3)
C7—C8—H8B	109.8	C18—C19—H19	119.3
C3—C8—H8B	109.8	C14—C19—H19	119.3
			
C1—N1—N2—C2	−0.20 (19)	C2—C3—C8—C7	179.05 (15)
C13—N1—N2—C2	−172.34 (16)	C9—C3—C8—C7	−59.69 (19)
N2—N1—C1—O1	0.44 (19)	C4—C3—C8—C7	59.4 (2)
C13—N1—C1—O1	172.06 (15)	C2—C3—C9—C10	−178.88 (14)
N2—N1—C1—S1	179.88 (13)	C4—C3—C9—C10	−59.16 (18)
C13—N1—C1—S1	−8.5 (3)	C8—C3—C9—C10	59.75 (19)
C2—O1—C1—N1	−0.49 (17)	C3—C9—C10—C11	60.0 (2)
C2—O1—C1—S1	−179.97 (13)	C3—C9—C10—C12	−60.2 (2)
N1—N2—C2—O1	−0.13 (18)	C9—C10—C11—C5	−60.5 (2)
N1—N2—C2—C3	177.74 (16)	C12—C10—C11—C5	60.0 (2)
C1—O1—C2—N2	0.40 (19)	C4—C5—C11—C10	60.3 (2)
C1—O1—C2—C3	−177.71 (14)	C6—C5—C11—C10	−59.6 (2)
N2—C2—C3—C9	9.1 (2)	C6—C7—C12—C10	60.7 (2)
O1—C2—C3—C9	−173.15 (14)	C8—C7—C12—C10	−60.1 (2)
N2—C2—C3—C4	−110.8 (2)	C9—C10—C12—C7	59.9 (2)
O1—C2—C3—C4	66.93 (19)	C11—C10—C12—C7	−60.5 (2)
N2—C2—C3—C8	129.51 (19)	C14—N3—C13—N1	90.8 (2)
O1—C2—C3—C8	−52.7 (2)	C1—N1—C13—N3	87.8 (2)
C2—C3—C4—C5	179.79 (16)	N2—N1—C13—N3	−101.2 (2)
C9—C3—C4—C5	59.2 (2)	C13—N3—C14—C15	−15.3 (3)
C8—C3—C4—C5	−59.6 (2)	C13—N3—C14—C19	165.89 (18)
C3—C4—C5—C11	−59.9 (2)	N3—C14—C15—C16	−178.7 (2)
C3—C4—C5—C6	60.3 (2)	C19—C14—C15—C16	0.1 (3)
C4—C5—C6—C7	−60.4 (2)	C14—C15—C16—C17	0.9 (4)
C11—C5—C6—C7	59.5 (2)	C15—C16—C17—C18	−0.9 (4)
C5—C6—C7—C12	−60.2 (2)	C16—C17—C18—C19	0.0 (4)
C5—C6—C7—C8	60.7 (2)	C17—C18—C19—C14	1.0 (3)
C12—C7—C8—C3	60.39 (19)	N3—C14—C19—C18	177.80 (19)
C6—C7—C8—C3	−60.3 (2)	C15—C14—C19—C18	−1.0 (3)

### Hydrogen-bond geometry (Å, º)

Cg1 is the centroid of the C14–C19 ring.

**Table:**

*D*—H···*A*	*D*—H	H···*A*	*D*···*A*	*D*—H···*A*
N3—H3···S1^i^	0.87 (1)	2.62 (1)	3.4763 (19)	170 (2)
C13—H13*A*···*Cg*1^ii^	0.97	2.83	3.549 (2)	132

Symmetry codes: (i) −*x*+1/2, *y*−1/2, −*z*+3/2; (ii) −*x*+1, −*y*+1, −*z*+2.
